# Advances in the Management of Mandibular Fractures: A Comprehensive Review of Surgical Techniques and Biomaterials

**DOI:** 10.7759/cureus.107942

**Published:** 2026-04-29

**Authors:** Nishita Rajan Kadu, Ruchi Mitra, Mihir Patel, Srushti Shah, Bhavani K, Anjalina Jena

**Affiliations:** 1 Department of Dental Surgery, Mahatma Gandhi Mission (MGM) Dental College and Hospital, Navi Mumbai, IND; 2 Department of Dentistry, Phulo Jhano Medical College and Hospital, Dumka, IND; 3 Department of Dentistry, Manubhai Patel Dental College, Vadodara, IND; 4 Department of Dentistry, Goenka Research Institute of Dental Science, Gandhinagar, IND; 5 Department of Microbiology, Sree Balaji Medical College and Hospital, Chennai, IND; 6 Department of Dentistry, Kalinga Institute of Dental Sciences, Bhubaneswar, IND

**Keywords:** biomaterials, bone grafts, mandibular fractures, maxillofacial trauma, three-dimensional printing

## Abstract

Mandibular fractures represent a significant component of maxillofacial trauma, frequently associated with functional impairment, esthetic compromise, and complex biomechanical challenges. Advances in imaging, surgical techniques, and biomaterials have progressively transformed management strategies, shifting emphasis toward stable fixation, early mobilisation, and biologically favourable healing. The objective of this narrative review is to synthesise contemporary evidence on evolving surgical approaches and biomaterial applications in mandibular fracture management, highlighting clinical outcomes and emerging trends. A comprehensive literature search was conducted across major electronic databases, including PubMed/MEDLINE (Public Medline/Medical Literature Analysis and Retrieval System Online), Scopus, Web of Science, and Google Scholar, encompassing publications from 2015 to 2025. Eligible studies included clinical investigations and reviews addressing diagnostic advances, fixation techniques, biomaterials, and technological innovations relevant to mandibular fractures. The review integrates current concepts in fracture classification, closed and open reduction techniques, advances in plate and screw systems, resorbable and bioactive materials, bone grafts and substitutes, minimally invasive approaches, and digital technologies such as virtual surgical planning and three-dimensional printing. Contemporary evidence supports individualised treatment planning based on fracture morphology, biomechanical demands, and patient-specific factors. Modern mandibular fracture management reflects a multidisciplinary and technology-driven paradigm that prioritises anatomical accuracy, functional restoration, and reduced morbidity. Continued integration of advanced biomaterials and digital workflows is expected to further refine clinical outcomes in maxillofacial trauma care.

## Introduction and background

Mandibular fractures are among the most common maxillofacial injuries and contribute substantially to emergency department admissions and surgical workload worldwide; recent data from India show that road traffic accidents remain the leading cause of maxillofacial trauma (53.5%), and mandibular fractures account for 38% of all facial fractures in a large North Indian series, while mortality remains low in isolated mandibular fractures but may increase in the presence of associated polytrauma [1]. The most common etiological factors are road traffic accidents, interpersonal violence, falls, and sport-related injuries, and regional variation depends on the socioeconomic and environmental conditions [2]. The prominence, mobility, and horseshoe-shaped anatomy of the mandible make it especially vulnerable to trauma. Fractures commonly occur at anatomically weak sites such as the symphysis, parasymphysis, body, angle, ramus, condyle, and coronoid process [3,4]. Investigations in the field of imaging, such as computed tomography and cone-beam computed tomography, have improved the accuracy of diagnosis and allowed to examine the patterns of fractures and displacement in greater detail [5]. The mandible plays a crucial role in maintaining lower facial contour, dental occlusion, mastication, speech, and airway stability [6]. Disruption of mandibular continuity may result in malocclusion, restricted movement, chronic pain, neurosensory deficits, and impaired facial esthetics, thereby significantly affecting function and quality of life [7]. Reestablishment of anatomical position and occlusal harmony continues to be the focus of successful treatment, as well as maintenance of mandibular biomechanics and reduction of long-term morbidity [8]. Management of mandibular fractures remains clinically challenging because it involves complex interactions between fracture segments, dentition, masticatory muscles, and the inferior alveolar neurovascular bundle [9]. The microbial environment of the oral cavity predisposes open fractures to infection, particularly in cases of comminution or delayed treatment [10]. Surgical site infection, malunion, non-union, malocclusion, plate exposure and hardware failure have been reported as complications, and each could lead to compromised functional recovery [11].

Patient-specific factors such as systemic disease, nutritional status, tobacco use, and adherence to postoperative care protocols significantly influence healing outcomes and complication rates [12]. Traditional management techniques relied heavily on closed reduction and prolonged intermaxillary fixation [13]. These procedures are effective in the selected fracture patterns, but they were also linked with a prolonged immobilisation period, a decrease in airway safety, nutritional problems, and patient discomfort [7]. Historically, mandibular fracture management relied predominantly on closed reduction techniques and prolonged intermaxillary fixation, which were associated with functional limitations and patient discomfort [4]. However, recent decades have witnessed significant advancements with the introduction of open reduction and internal fixation, improved plate and screw systems, resorbable biomaterials, and digital technologies such as virtual surgical planning and three-dimensional printing, leading to enhanced anatomical accuracy, early functional recovery, and improved clinical outcomes [10]. The development of open reduction and internal fixation represented a major advancement by enabling early mobilisation and precise anatomical restoration [3]. The introduction of miniplate osteosynthesis, locking plate systems, as well as the concept of load-sharing fixation, permitted the management of fractures that were stable without the need to rely on maxillomandibular fixation, which facilitated functional rehabilitation [14]. Concurrent advances in biomaterial science have had a major say in modern mandibular fracture repair [11]. The fixation systems made of titanium are predictably strong and biocompatible, although issues of stress shielding, retention of implants over time, palpability, and radiographic artefacts remain [15]. Such restrictions have prompted the creation of resorbable fixation, magnesium and alloy and bioactive materials aimed at facilitating the growth of the bone and slow loading transfer [16]. Synthetic substitutes and bone grafts have become more and more relevant in the treatment of comminuted fractures, segmental defects and complicated cases with infection or bone loss to increase the range of reconstructive choices in complicated clinical cases [8]. Surgical planning and execution have been further refined by technological innovation [2]. Three-dimensional visualisation, virtual surgical planning, and additive manufacturing now enable precise preoperative simulation and the fabrication of patient-specific implants and surgical guides [17]. These computer tools have shown possibilities of enhancing accuracy in fracture reduction, cutting time, and postoperative symmetry and occlusal results [18]. Despite increasing clinical use, access, cost-effectiveness, and standardised implementation remain inconsistent across healthcare systems [13]. The heterogeneity in treatment choice, fixation methods and biomaterial usage still exists despite the broad development. Available literature often covers each of the techniques or materials separately, which makes it difficult to compare the results and profile of complications. Rapid innovation has outpaced the creation of coherent clinical guidelines, which leaves ambiguity over the best practices to use in managing this or that fracture or patient group. An in-depth analysis of the modern surgical methods and biomaterials is necessary to unify the existing evidence and stimulate evidence-based clinical decision-making in the management of mandibular fractures.

Objectives of the review

The objective of this review is to synthesise current evidence on advances in the surgical management of mandibular fractures, with focused emphasis on evolving fixation techniques and biomaterials. Clinical outcomes, limitations, and emerging technologies are critically examined to inform contemporary maxillofacial practice.

Methodology

A narrative literature search was conducted across major electronic databases, including PubMed/MEDLINE, Scopus, Web of Science, and Google Scholar, to identify relevant studies on mandibular fracture management. The search strategy employed structured keyword combinations and Boolean operators (AND, OR), including terms such as “mandibular fractures,” “maxillofacial trauma,” “open reduction and internal fixation,” “biomaterials,” “resorbable fixation,” “bone grafts,” and “three-dimensional surgical planning.” Studies published between 2015 and 2025 were considered for inclusion.

Retrieved records were screened in a stepwise manner, beginning with title and abstract screening for relevance, followed by full-text evaluation of potentially eligible studies based on predefined inclusion and exclusion criteria. Reference lists of selected studies were manually reviewed to identify additional relevant studies. Only studies published in English were included to ensure consistency in data interpretation. Studies eligible for inclusion comprised original clinical investigations, systematic reviews, meta-analyses, and narrative reviews addressing surgical management, fixation techniques, or biomaterials used in the treatment of mandibular fractures in human subjects. Studies reporting clinical outcomes, complication profiles, biomechanical considerations, or recent technological developments were considered relevant.

Studies consisting primarily of case reports, experimental animal studies, in vitro investigations, conference abstracts, editorials, or those lacking sufficient methodological rigour or clinical relevance were excluded. Literature focusing on isolated dental trauma, pathological fractures unrelated to trauma, or fractures involving facial bones other than the mandible was not included as a primary focus of this review. Given the narrative design of this review, formal risk-of-bias assessment and quantitative synthesis were not performed. Relevant findings were synthesised qualitatively to provide a concise overview of current evidence and recent developments in mandibular fracture management. This review followed a narrative approach; therefore, statistical pooling, meta-analysis, and quantitative comparisons were not performed due to heterogeneity in study designs, fracture types, and outcome measures. Accordingly, statistical indicators such as effect sizes, confidence intervals, and P-values are not applicable. No artificial intelligence-based tools were utilised in the literature search, screening, or data extraction process.

## Review

Classification and diagnostic advances in mandibular fractures

Accurate classification of mandibular fractures is essential for diagnosis, treatment planning, and outcome prediction [18]. Modern classification produces differentiation of fractures according to place of location, fracture pattern, displacement, favorability, and status of dentition [19]. Anatomical classification is still in use and categorises fractures as symphyseal, parasymphyseal, body, angle, ramus, condylar, and coronoid [14]. This intervention helps clinicians communicate and helps them select surgical access and fixation strategies [20]. Fractures are also further classified as either favourable or unfavourable according to the tendencies of muscles to pull and displace, and this offers a clue to the biomechanical stability and fixation demands [9]. Classification based on fracture patterns has gained importance with the increasing recognition of comminuted, greenstick, and pathological fractures [15]. Comminuted fractures have special issues with regard to stability of fragments and vascular compromise, which can lead to the requirement of rigid fixation or reconstruction plates [21]. Classifications based on dentition, which differentiate fractures in either dentate, partially dentate, or edentulous mandibles, have an impact on load-bearing factors and choice of implants [7]. These categories favour personalised management, especially among older patients and when patients have atrophic mandibles [22].

The development of diagnostic imaging has greatly enhanced the assessment of fractures, which was previously done by the use of conventional radiography [13]. Computer tomography has replaced the diagnostic criteria of mandibular trauma as it has excellent spatial resolution facets and multiplanar reconstruction properties [16]. Great sensitivity in identifying lowly displaced fractures, condylar injuries, and comminution has curtailed diagnostic confidence and missed traumas [12]. Compared to other methods, cone-beam CT has similar diagnostic quality with a reduced dose of radiation and, therefore, is becoming more pertinent in cases of dentoalveolar and isolated mandibular trauma [23]. The diagnostic processes have also changed because of three-dimensional imaging and reconstruction technologies [11]. Three-dimensional visualisation improves understanding of fracture morphology, spatial displacement, and occlusal relationships [6]. Virtual surgical planning can be integrated to simulate fracture reduction and fixation in preoperative models, increasing the accuracy of complex or comminuted injuries [18]. These digital innovations improve surgical accuracy, reduce intraoperative uncertainty, and support better postoperative outcomes, particularly in anatomically complex regions such as the condyle and mandibular angle [24]. The development of classification systems and the development of the high-quality imaging modalities has reinforced the accuracy of diagnosis and predictability of treatment [7]. Modern methods focus on the full description of fractures and allow surgeons to develop individual surgical techniques that suit the biomechanical requirements and patient characteristics [19]. Table 1 shows the current classification criteria and diagnostic modalities that are used in the assessment of mandibular fractures.

**Table 1:** Classification Parameters of Mandibular Fractures and Their Clinical Significance CT: Computed Tomography, CBCT: Cone-Beam Computed Tomography, 3D: Three-Dimensional

Classification Parameter	Description	Clinical Relevance	Common Examples	Diagnostic Modality	References
Anatomical Location	Categorisation by mandibular region	Guides surgical access and fixation choice	Symphysis, angle, condyle	CT, CBCT	Parente Arias et al., 2016 [23]
Fracture Pattern	Based on fragment configuration	Influences stability and fixation rigidity	Simple, comminuted	CT	Giovannetti et al., 2019 [18]
Displacement	Degree and direction of fragment movement	Predicts the need for open reduction	Displaced, non-displaced	CT, 3D imaging	Shenoi et al., 2024 [20]
Favorability	Relation to muscle pull	Determines fixation strategy	Favorable, unfavorable	CT	Bansal et al., 2021 [13]
Dentition Status	Presence or absence of teeth	Affects the load-bearing approach	Dentate, edentulous	CT, CBCT	Stathopoulos et al., 2024 [17]

Principles of mandibular fracture management

Management of mandibular fractures is guided by three core principles: anatomical reduction, stable fixation, and early functional recovery [25]. The objectives of these principles are to restore mandibular continuity, ensure occlusal harmony, and retain neuromuscular activity [19]. Sufficient reduction provides the correct location of the fracture pieces, and the possibility of preventing malocclusion and dysfunction of the temporomandibular joint is minimised [11]. As compared to long-term maxillomandibular immobilisation, stable fixation aids bone healing during functional loading and decreases dependency [22]. The Arbeitsgemeinschaft für Osteosynthesefragen (AO) principles have continued to be a significant guiding principle in the treatment of mandibular fractures [17]. These principles include anatomical reduction, constant fixation of the internal organs, maintenance of blood flow, and early mobility [26]. Due to the need to respect periosteal and endosteal vascularity during surgical exposure, the results would promote the best healing and decrease the risk of infection or delayed union [18]. Early functional rehabilitation stimulates physiological bone remodelling and avoids the disuse-related problems, including muscle atrophy and joint stiffness [22]. Biomechanical factors are very important in determining proper fixation techniques [18].

**Figure 1:**
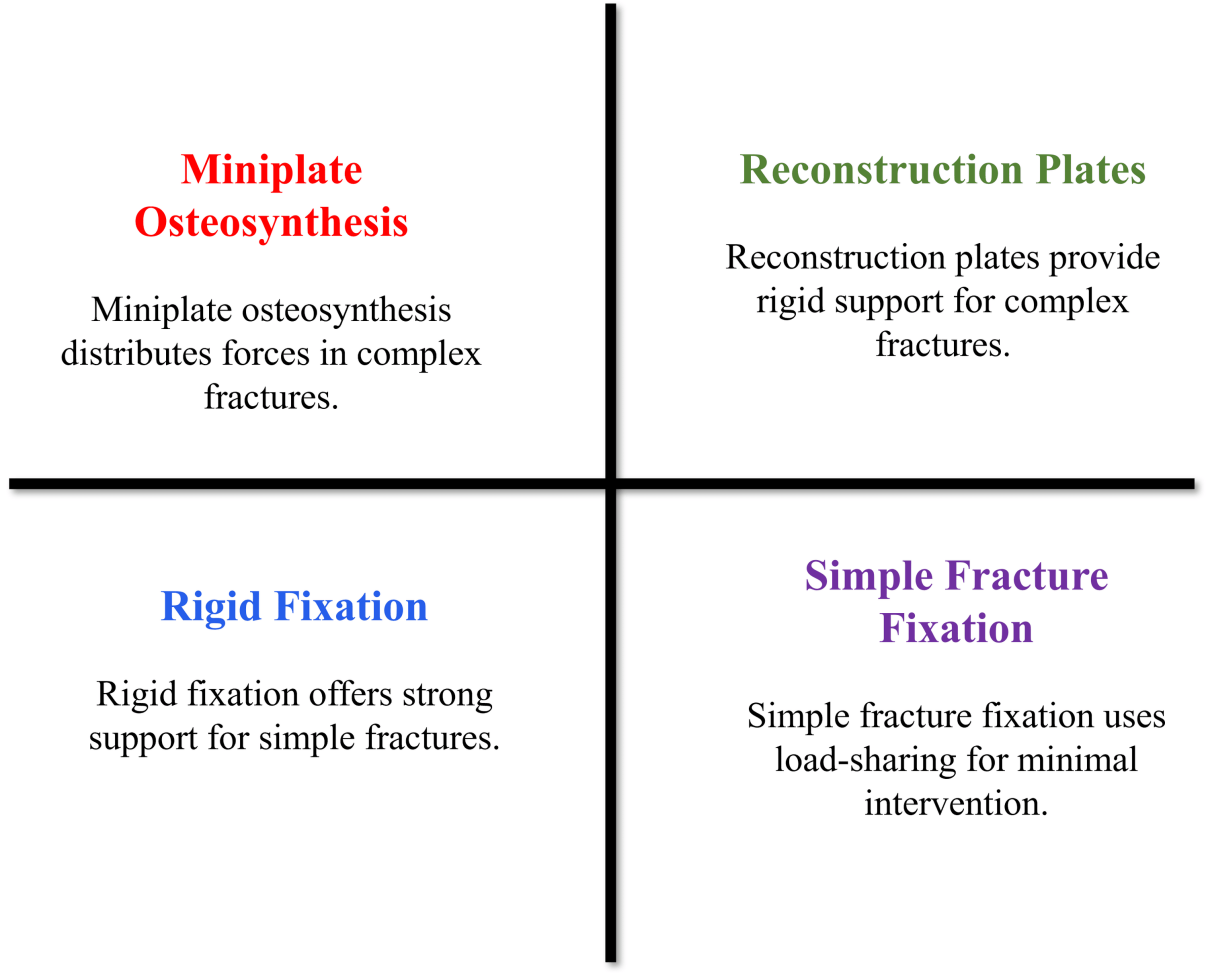
Mandibular Fracture Fixation Strategies Created by authors using Microsoft PowerPoint.

The complex forces experienced by the mandible are tension, compression, torsion, and bending forces produced by the process of mastication [27]. Understanding force distribution along the mandibular arch helps guide implant placement and fixation design [14]. Compressive stresses are mostly found in areas like the inferior border, and tensile stresses are found in the alveolar region during functioning [21]. The concepts of fixation can be classified into the load-bearing and the load-sharing approaches [16]. Load-bearing fixation transfers the majority of functional forces to the fixation device and is typically used in comminuted fractures, atrophic mandibles, and segmental defects [26]. An example of this technique is reconstruction plates, which offer rigid fixation through fracture locations [20]. The fixation of load-sharing allocates the masticatory forces between the bone and implant, which is appropriate to simple fractures that have sufficient bony contact [7]. Miniplate osteosynthesis still finds large use in these situations, and does not cause excessive hardware rigidity to support functional loading [21]. Alignment with biomechanical principles allows treatment to be tailored to different fracture patterns, thereby improving stability, healing, and functional recovery [17]. Figure 1 shows how the selection of fixation technique is guided by fracture complexity, stability requirements, and the balance between mechanical support and biologic preservation.

Closed reduction techniques: current perspectives

Closed reduction remains a useful option for selected mandibular fractures, particularly those with minimal displacement and preserved occlusal relationships [27]. It is based on indirect fixation of fractures without the need to expose the patient and achieve anatomical position with external or intraoral immobilisation techniques [28]. The typical signs are non-dislocated or minimally dislocated fractures, desirable fracture lines, childhood greenstick fractures, and medically impaired patients, in whom surgery is more hazardous [22]. In clinical practice, closed reduction is primarily based on intermaxillary fixation. Intermaxillary fixation fixes fracture segments and promotes bone healing by controlled functional rest by immobilising the mandible with respect to the maxilla [29]. The traditional techniques are arch bars, eyelet wiring, and Ivy loops, which offer good guidance to the occlusion and stabilisation [5]. The progress in fixation devices has also provided intermaxillary fixation screws, which require less time to apply, are more comfortable to patients, and have better oral hygiene preservation properties than the conventional wiring methods [30].

Closed reduction has a number of benefits, such as no surgical exposure, maintenance of periosteal blood flow, shortened time of operation, and decreased occurrence of surgical site infection [24]. These advantages make the method especially adequately fit in resource-constrained environments and emergency response [17]. Fracture hematoma and soft tissue attachments further assist in healing processes that are biological in nature [26]. Functional outcomes tend to improve in the right cases, especially in cases where pre-injury occlusion has been restored [31]. After all, despite its usefulness, closed reduction is linked with quite significant limitations. The prolonged immobilisation can lead to diminished airway safety, nutritional difficulties, weight loss, and diminished patient compliance [29]. Stiffness of the temporomandibular joint, muscle atrophy, and poor oral hygiene are other issues [16]. Poor fracture stability can lead to malunion or chronic malocclusion, particularly in poor or comminuted fracture morphologies [30]. These limitations have gradually reduced the use of closed reduction in favour of rigid internal fixation for more complex injuries [32]. The contemporary views focus on the selective use of closed reduction in relation to the morphology of the fracture, the factors of the patient, and the functional requirements [33]. Intermaxillary fixation protocols have been proposed in the short-term as a method to reduce the morbidity associated with immobilisation in conjunction with hybrid methods that involve closed reduction with additional stabilisation [27]. Prudent patient selection and observance of biomechanical principles are also important towards maximisation of results [15]. Table 2 outlines commonly employed closed reduction techniques, their indications, advantages, and limitations in contemporary mandibular fracture management.

**Table 2:** Intermaxillary Fixation Techniques in Mandibular Fracture Management IMF: Intermaxillary Fixation

Technique	Fixation Method	Primary Indications	Advantages	Limitations	References
Arch bars	Circumdental wiring	Dentate, minimally displaced fractures	Reliable occlusal control	Oral hygiene difficulty	Boffano et al., 2015 [25]
Eyelet wiring	Wire loops around teeth	Simple fracture patterns	Cost-effective	Limited stability	Shenoi et al., 2024 [20]
Ivy loops	Interdental wiring	Short-term immobilization	Rapid application	Technique sensitivity	Jain and Rai, 2021 [31]
IMF screws	Bone-anchored screws	Adult, cooperative patients	Improved comfort	Root injury risk	Chen et al., 2016 [27]

Open reduction and internal fixation (ORIF): evolution of techniques

Open reduction and internal fixation has become a cornerstone of modern mandibular fracture management because it enables precise anatomical reconstruction and early functional recovery [29]. The initial methods were based on transosseous wiring, which offered low levels of stability and often necessitated longer intermaxillary fixation to stabilise the fractures [34]. These procedures were linked to increased cases of malocclusion, delayed union, and patient discomfort, especially in fractures experiencing a big masticatory force [30]. The introduction of rigid internal fixation marked a major advance in maxillofacial trauma care. Mini plate osteosynthesis, which was developed based on the biomechanical principles, allowed the fixation to be conducted in a stable position along the optimal lines of osteosynthesis without overexposing the surgery [26]. This change has made fracture-site functional loading feasible and minimised the necessity of extended maxillomandibular immobilisation [35].

Locking plate was another technology that increased the fixation stability by reducing the compression between plate and bone, improving the blood flow to periosteal and enhancing the osteoporotic or comminuted bone performance [33]. The indications of ORIF are displaced fractures, poor fracture type, fracture sites, comminuted injuries, and malocclusion/functional fractures [23]. ORIF is also preferred in instances where the mandibular functions need to be returned early, airways secured, and the patient made more comfortable [20]. Angular, body, and symphysis fractures are often operated on through open methods because the fractures are accessible and have foreseeable fixation results [34]. Condylar fracture, which was previously treated conservatively, has been undergoing ORIF in a few instances to regain vertical height and mandibular symmetry [15]. ORIF is associated with improved occlusal stability, lower morbidity, and better quality of life [35]. Mobilisation at an early stage helps in physiological bone healing and minimizes the chances of stiffness in the temporomandibular joint and atrophy of muscles [31]. Such complications as infection, plate exposure, and hardware failure remain relatively low when proper surgical technique and the choice of fixation are used [36]. The development of ORIF procedures is still advancing the treatment of mandibular fractures to achieve increased stability, biological retention, and restoration of functions in accordance with the current surgical practices [36].

Biomaterials and fixation strategies in mandibular fracture management

Plate and Screw Fixation Systems

The development of plate and screw fixation systems has significantly improved the predictability of mandibular fracture management [22]. The first fixation appliances were restrained by poor rigidity and relied on long-term maxillomandibular fixation [30]. Modern systems permit stable osteosynthesis under functional loading conditions while respecting mandibular biomechanics and fracture characteristics [36]. Miniplate osteosynthesis has continued to be used in simple and desirable fracture patterns [33]. When placed along ideal osteosynthesis lines, these systems distribute load effectively between bone and implant and support early mobilisation [37]. Designs made with a low profile result in less irritation of soft tissues and lower palpability, especially in the anterior mandible [34]. Titanium miniplates have good biocompatibility and corrosion resistance, which leads to foreseeable healing results and few complications [38].

Locking plate systems provide a fixed-angle construct that enhances stability without requiring close plate-to-bone contact, thereby preserving periosteal blood supply and reducing screw loosening [35,39]. These systems are particularly useful in comminuted fractures and poor-quality bone [40]. Reconstruction plates are indicated in load-bearing situations such as segmental defects and atrophic mandibles, where they provide rigid structural support under high functional forces [21,27]. Recent advances in contouring, including prefabricated and patient-specific plates, have further improved adaptation and reduced intraoperative manipulation [17].

**Figure 2:**
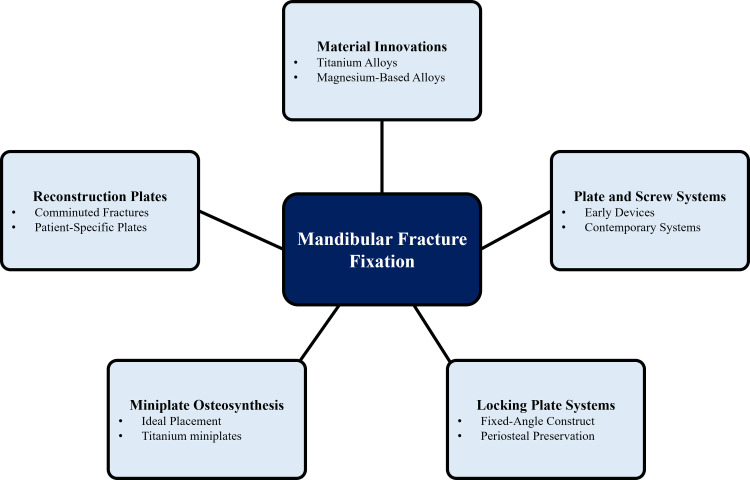
Advances in Mandibular Fracture Fixation Created by authors using Microsoft PowerPoint.

Fixation performance has been further refined with the help of material inventions. The development of new materials, such as magnesium-based alloys and surface-modified implants, is supposed to increase the osteointegration process and decrease the long-term implant retention [41]. Optimisation of the biomechanical properties of plate thickness, screw diameter, and hole design is still in progress to enhance resistance to torsion and bending forces during the mastication process [22]. All these developments facilitate stable fixation, biological preservation, and better clinical results in the fracturing of the mandible [42]. In addition to metallic fixation systems, increasing attention has been directed toward biomaterials that enhance biological healing. Figure 2 shows how these developments enable tailored fixation strategies by integrating biomechanical stability, biologic preservation, and fracture-specific requirements.

Resorbable and Bioactive Biomaterials

Resorbable and bioactive biomaterials have gained increasing interest as alternatives to permanent metallic fixation systems in the management of mandibular fractures [43]. These materials provide temporary mechanical support during bone healing and gradually degrade over time, thereby eliminating the need for secondary implant removal surgery [39]. They are particularly useful in pediatric patients, esthetically sensitive areas, and situations in which long-term implant retention may be undesirable [41]. Removable fixation systems are usually made of polymers like polylactic acid, polyglycolic acid, and copolymers of each [35]. These materials have positive biocompatibility and degradation characteristics that are predictable under physiological conditions [44]. Advances in polymer engineering have improved mechanical strength and reduced inflammatory reactions compared with earlier generations of resorbable implants [27]. Gradual loss of functional load to regenerative bone is facilitated by controlled degradation and helps physiological remodelling, and limits the action of stress shielding [45].

Unlike passive fixation materials, bioactive biomaterials interact directly with the surrounding biological environment [46]. Bioactive-based materials that include bioactive ceramics, calcium phosphate compounds, or surface-modified polymers, promote osteoconduction and improve bone healing at fracture sites [37]. These materials support cellular adhesion, proliferation, and mineralisation, thereby improving integration between the implant and host bone [42]. The properties are especially beneficial in fractures that are complicated by bone loss or impaired healing capacity [47]. Evidence of clinical outcomes of resorbable and bioactive systems depicts satisfactory stability of fracture, good complication rates, and good functional recovery in some selected cases [31]. Less risk of long-term palpability, imaging interference, and restriction of growth in pediatric mandibles is reported [40]. Limitations are still connected with lower initial mechanical strength than titanium systems, thermal and mechanical handling sensitivity, and increased costs of materials. The selection of cases and the principle of biomechanics must be strictly followed to maximise clinical success [39]. Further improvement in material composition, degradation behavior, and bioactivity is targeted to increase the clinical usability of resorbable and bioactive biomaterials. Their increasing use reflects a broader shift toward biologically driven fixation strategies that support healing, function, and patient-centred outcomes. These developments are further complemented by the use of bone grafts and substitutes in cases involving bone loss or compromised healing.

Bone Grafts and Bone Substitutes

Bone grafts and substitutes are used in mandibular fractures to restore structural continuity and support bone regeneration in cases with defects or bone loss [48]. The signs are comminuted fractures, segmental defects, infected non-unions, atrophic mandibles, and bone loss-related fractures [23]. These materials promote osteogenesis, provide structural support, and facilitate stable healing by filling defects and stimulating new bone formation at damaged sites [39].

The autogenous bone grafts are considered the reference standard in mandibular restoration because of the inherent osteogenic, osteoinductive, and osteoconductive properties [35]. Typical sites of donor include the iliac crest, mandibular symphysis, ramus, and calvarium. Their high biocompatibility supports predictable remodelling and integration without eliciting an immune response [42]. Limitations are donor site morbidity, longer operative time, and small graft volume, which could limit its application in large defects [27].

The alternative options are allografts and xenografts that help to avoid the morbidity of the donor sites and provide more accessibility [30]. These grafts are mainly operative by osteoconduction, which acts as a scaffold for the growth of the host bones. Immunogenicity and risk of transmission of diseases are decreased by advanced processing methods, which increase safety profiles [27]. There are slower incorporation rates and lower biological activity than autografts, which are still significant limitations, especially in load-bearing mandible areas [45]. Synthetic bone replacements have been used because of a stable supply, a versatile nature, and a lack of transmission of diseases [44]. Hydroxyapatite, beta-tricalcium phosphate, and bioactive glass are some of the materials that portray desirable osteoconductive properties [39]. A combination of ceramics with polymers or systems to deliver growth factors are composite biomaterial that is expected to improve mechanical stability and biological performance. They can be used to replace contained defects and as graft extenders with autogenous bone [21].

The choice of graft material depends on defect size, complexity of the fracture, biomechanical requirements, and also patient factors are considered [29]. Bone grafts are integrated with rigid fixation systems, which help in structural stability and functional rehabilitation [46]. Emerging approaches include bioengineered grafts, scaffold-based regeneration, and biologically active composites designed to enhance healing and reduce morbidity [42]. Table 3 provides a comparative overview of commonly used bone grafts and bone substitutes in mandibular fracture repair.

**Table 3:** Bone Graft Options in Mandibular Fracture Repair

Graft Type	Source	Biological Properties	Advantages	Limitations	References
Autograft	Patient-derived bone	Osteogenic, osteoinductive, osteoconductive	High integration potential	Donor site morbidity	Gillman and Jayasuriya, 2021 [47]
Allograft	Human donor bone	Osteoconductive	No second surgical site	Slower incorporation	Kamath et al., 2023 [43]
Xenograft	Animal-derived bone	Osteoconductive	Structural stability	Limited remodeling	Demirdöver and Geyik, 2024 [39]
Synthetic graft	Man-made materials	Osteoconductive	Unlimited supply	Lower biological activity	Suojanen et al., 2016 [42]

Minimally invasive and endoscopic-assisted techniques

Less invasive and endoscopic-assisted surgeries have become useful in the treatment of selected mandibular injuries to decrease the morbidity of surgery and improve functional outcomes [38]. These methods emphasise minimal surgical exposure, soft tissue integrity, and periosteal blood supply [40]. It is especially applicable in fractures of the condylar region, subcondylar region, and mandibular ramus, in which traditional open techniques are also linked to the high chances of facial nerve injury and visible scarring [46]. Endoscopic-assisted reduction provides the possibility to view the fracture sites directly with the help of a small intraoral or transbuccal opening [48]. Opticals with high resolution ensure the endoscopy offers views of tissues with magnification, as this makes it easy to reduce the fracture and fixation device placement accurately [28]. Enhanced visualisation assists in the restoration of mandibular height, alignment, and occlusal relationship, more so in displaced condylar fracture [20]. Such methods provide the ability to fix the interior in a stable position and with a minimum of expensive dissectors to achieve better anatomical results [41].

Minimally invasive procedures have a number of clinical benefits. Less trauma of the soft tissues is linked to lower oedema anaesthetics, reduction of pain and infection [38]. The maintenance of muscular attachments assists in the early mobilisation of the mandible and rehabilitation [47]. Reduced length of stay and faster recovery to daily activities have been noted, thus improving patient satisfaction and the general quality of care [41]. Less external scarring also leads to good esthetic outcomes, particularly in the younger patients and those who have a great concern about cosmetic appearance [45]. In spite of such advantages, endoscopic-assisted and minimally invasive methods have technical challenges [34]. The high learning curve, special equipment, and the complexity of the operation could be inhibitory to its use [30]. Poor visualisation of severely comminuted breaks or where there is a high level of displacement can affect the accuracy of reduction. Proper selection of patients, experience of the surgeons, and following of the biomechanical principles are still crucial in realising the best results [47]. The further development of endoscopic apparatus, imaging systems, and fixation systems is then likely to broaden the scope of the minimally invasive methods [43]. Their application in the management of mandibular fractures is symptomatic of the broader trend of adopting tissue-saving surgical approaches to mandible fracture, favouring a focus on functional restoration and minimised morbidity and patient-focused outcome.

Digital technologies and 3D printing in mandibular fracture management

Digital technologies have reshaped mandibular fracture management by improving diagnostic accuracy, surgical planning, and intraoperative efficiency [48]. With the help of the advanced imaging modalities, the computer-aided design and production processes make it possible to visualise the morphology of fractures and to analyse the spatial relationships in detail [37]. The technologies assist in personalised treatment plans that match the anatomical restoration and conform to biomechanical needs, especially in intricate and comminuted fracture designs [42]. Virtual surgical planning has become an important component of contemporary maxillofacial trauma care [36]. The data provided by high-resolution computed tomography enables three-dimensional reconstruction of the mandible, which makes it easier to accurately determine fracture displacement, the position of the segments, and the relationships between the occlusiveness [27]. In virtual simulation, preoperative assessment of the position of implants, screw paths, and plate contouring is allowed [39]. This method aids in making the surgery more predictable, decreases the variability in intraoperative decisions, and assists in the effective completion of the complicated surgery [44].

The three-dimensional printing technology makes the virtual planning of the surgical solutions a reality. Patient-specific surgical guides help in the correct placement of osteotomy, reduction of fractures, and placement of implants, and reduce the intraoperative error [46]. Individualised fixation plates produced by additive manufacturing offer specific anatomical alignment of bone segments and lessen intraoperative bending, as well as minimise operative time [48]. Better implant fit is associated with stability, decreased soft tissue irritation, and maximum load distribution at fracture locations [41]. Digital workflow clinical integration has been shown to be beneficial in the reduction of operative time, better anatomical accuracy, and postoperative functionality and esthetics [35]. Such benefits are especially notable in instances of mandibular fractures that have asymmetry, more than one line of fracture, or already existing deformities [27]. These tools also support preoperative patient education and interdisciplinary communication, thereby strengthening comprehensive treatment planning [38]. The continued development of software platforms, printing material, and workflow integration will enhance the cost-effectiveness and expand clinical applicability [33]. The use of digital technologies and 3D printing is a breakthrough in the management of mandibular fractures, which facilitates patient-centred, precision-guided surgery.

Complications, outcomes, and evidence-based comparisons

Complications remain an important determinant of treatment selection and long-term outcomes in mandibular fracture management [48]. The problem of postoperative infection has remained common, especially in fractures in close contact with the mouth [42]. Late intervention, large-scale soft tissue damage, impaired vascularity, and poor stabilisation are some of the factors that predispose a person to infection [37]. Another major complication is malocclusion, which is found to mostly occur due to improper reduction of the fracture or poor fixation stability [32]. Persistent occlusal imbalance may lead to temporomandibular joint dysfunction, impaired mastication, and ongoing pain [44]. Complications associated with hardware are exposure of the plate, loosening of screws, and fracture of the implant [46]. These occurrences are more commonly seen in those regions where high functional loading is exposed to or where poor bone quality is the case [30]. An inappropriate reaction of the plates and their over-rigidity when coupled with stress concentration can lead to fixation failure [36]. Neurosensory impairments of the inferior alveolar or mental nerves can take place following surgery, and will depend on the degree of nerve damage and surgical practice [47].

Outcome comparison studies have indicated unique benefits and shortcomings in different treatment modalities. The open reduction and internal fixation has proven to be better in displaced and undesirable fractures, providing better occlusal stability, shorter time to regain normal functioning, and diminished time of immobilisation [40]. The closed methods of reduction are useful in minimally displaced fractures and have positive results in terms of healing in cases of strict criteria of selection of cases [42]. The load-sharing fixation techniques demonstrate the lowest complication rates when simple fracture patterns are used, and the load-bearing systems are used to achieve a high level of stability in comminuted and atrophic mandibles [39]. Clinical outcomes are also affected by the selection of biomaterial. Titanium fixation systems are always found to be strong, durable, and predictable to heal [35]. The resorbable fixation systems have the benefit of reducing long-term morbidity of implants, especially in pediatric and esthetically sensitive sites, but the decreased initial mechanical strength restricts their use in high-load areas [48]. Synthetic substitutes and bone grafts help in enhancing the results of the fractures associated with bone loss, increasing the rate of union, and other aspects of the bone structure [42]. Available comparative evidence supports treatment planning based on patient-specific factors, biomechanical demands, and fracture morphology. The best results can be obtained by the selection of the methods of surgical techniques and biomaterials with reference to compliance with the biomechanical principles and thorough implementation of the surgeries. Ongoing production of comparative evidence is also a critical step in the refinement of the treatment algorithms and the enhancement of the outcome of mandibular fracture treatment.

Limitations and future recommendations

The scope of this review is subject to certain inherent limitations. Such limitations are as follows: high cost, use of special equipment, and reliance on technical competence. The level of accessibility is not constant in healthcare systems, and it affects the adoption rates [46]. The diverse study designs, fracture type, outcome measures, and follow-ups used in the studies included do not allow direct comparison and quantitative synthesis of the results. These differences in surgical experience, institutional procedures, and patient issues also affect reported outcomes. The use of mostly retrospective studies and narrative reviews decreases the strength of evidence in favour of a particular technique or biomaterial. It is also possible that publication bias and underreporting of complications would influence the completeness of the available data, especially with new technologies and new biomaterials.

Future research needs to focus on well-constructed prospective and multicenter studies with standardised classification systems and outcome assessment scales. Evidence-based decision-making would be enhanced by comparative trials that compare fixation methods and biomaterials according to certain fracture patterns. Clinical trials that evaluate the patient-reported measures, cost-effectiveness, and functional outcomes in the long term should be encouraged. The combination of regenerative, smart biomaterials and enhanced digital processes should be considered to optimise the management of mandibular fractures further and enhance patient-centred outcomes.

## Conclusions

Mandibular fracture management has advanced substantially through developments in surgical techniques, fixation systems, biomaterials, and digital technologies, leading to improved anatomical alignment and functional outcomes. Contemporary approaches emphasise accurate fracture reduction, biomechanically stable fixation, and early mobilisation to restore occlusion, mandibular continuity, and neuromuscular function. Advances in plate and screw designs, including locking and patient-specific systems, have enhanced stability across diverse fracture patterns. Increasing use of resorbable and bioactive biomaterials reflects a shift toward biologically compatible fixation that supports bone healing and reduces long-term implant-related morbidity. Bone grafts and synthetic substitutes remain important for complex fractures involving bone loss or impaired healing. Minimally invasive and endoscopic-assisted techniques offer tissue-sparing alternatives with potential reductions in morbidity and improved aesthetic outcomes. The integration of virtual surgical planning and three-dimensional printing has further improved diagnostic accuracy and surgical precision. Contemporary management increasingly favours an individualised approach based on fracture characteristics, biomechanical demands, and patient-specific factors. Continued technological innovation and evidence generation are essential for refining clinical practice and enhancing patient-centred outcomes in maxillofacial trauma care.
